# Alexithymia and Somatosensory Amplification Link Perceived Psychosocial Stress and Somatic Symptoms in Outpatients with Psychosomatic Illness

**DOI:** 10.3390/jcm7050112

**Published:** 2018-05-10

**Authors:** Mutsuhiro Nakao, Takeaki Takeuchi

**Affiliations:** 1 Department of Psychosomatic Medicine, School of Medicine, International University of Health and Welfare, Narita, Chiba 286-8686, Japan; 2 Department of Psychosomatic Medicine, Teikyo University Hospital, Tokyo 173-8605, Japan; takeakij@post.harvard.edu; 3 Department of Psychosomatic Medicine, School of Medicine, Toho University Hospital, Tokyo 143-8541, Japan

**Keywords:** alexithymia, anxiety, depression, excessive adaptation, psychosocial stress, psychosomatic medicine, somatic symptom, somatosensory amplification

## Abstract

Background: Psychosomatic patients often complain of a variety of somatic symptoms. We sought to clarify the role of clinical predictors of complaints of somatic symptoms. Methods: We enrolled 604 patients visiting a psychosomatic outpatient clinic. The outcome was the total number of somatic symptoms, and the candidate clinical predictors were perceived psychosocial stress, alexithymia, somatosensory amplification, adaptation, anxiety, and depression. All participants completed questionnaires assessing the outcome and the predictors. Results: The average number of reported somatic symptoms was 4.8; the most frequent was fatigue (75.3%), followed by insomnia (56.1%), low-back pain (49.5%), headache (44.7%), and palpitations (43.1%). Multiple regression analysis showed that the total number of somatic symptoms was significantly associated with the degree of perceived psychosocial stress, alexithymia, somatosensory amplification, and depression. Also, structural equation models indicated links between excessive adaptation (via perceived psychosocial stress, alexithymia, and somatosensory amplification) and the total number of somatic symptoms. Conclusion: The results suggested that the association between psychosocial stress and reported somatic symptoms is mediated by alexithymia and somatosensory amplification in psychosomatic patients.

## 1. Introduction

When people are vulnerable to stress because of inherent characteristics and their (in)ability to adapt, psychosomatic illness is likely to develop even if the stressors are mild or moderate [1]. The Japanese Society of Psychosomatic Medicine defines psychosomatic illness as any physical condition associated with organic or functional damage whose onset or development is affected by psychosocial factors [2]. Patients with psychosomatic illness often complain of a variety of somatic symptoms.

Alexithymia, a personality construct derived from clinical observations of patients with psychosomatic diseases, is characterized by difficulty distinguishing between emotions and bodily sensations [3]. The Toronto Alexithymia Scale (TAS) is one of the most common questionnaires used to measure this construct [4]. The evidence suggests that a tendency toward the development of functional somatic symptoms is associated with alexithymia [5,6]. Somatosensory amplification refers to the tendency to experience a somatic sensation as intense, noxious, and/or disturbing [7]. The construct of somatosensory amplification is helpful when assessing the perceptual style of somatization and in conceptualization of psychosomatic illness. The Somatosensory Amplification Scale (SSAS) was designed and validated to measure this phenomenon [8].

Both alexithymia and somatosensory amplification were found to affect somatic symptoms in our previous study [9], but other clinical factors (psychosocial adaptation and mood states) also seem to play roles in linking psychosocial stress and somatic symptoms. According to Selye’s stress theory of general adaptation syndrome [10], stress is a state produced by a change in the environment; adaptive coping contributes to resolution of the stress response, whereas maladaptive or excessively adaptive coping triggers further mind/body problems. A person preoccupied with perfect adaptation may subordinate his or her own needs to those of others and behave in a way prioritizing the needs of others [11]; appropriate adaptation to the environment is crucial in terms of stress management. Turning to mood states, persons with higher levels of depression and anxiety often visit psychosomatic clinics to report a variety of somatic symptoms [12].

Thus, our purpose in the present study was to confirm the roles played by alexithymia and somatosensory amplification as links between psychosocial stress and somatic symptoms. We hypothesized that alexithymia and somatosensory amplification were independently and positively associated with the reporting of somatic symptoms, even after controlling for other important variables (mood state and adaptive characteristics). To explore this hypothesis, the total number of somatic symptoms served as the outcome when we quantitatively and simultaneously assessed the effects of psychosocial stress, alexithymia, somatosensory amplification, depression, anxiety, and psychosocial adaption in outpatients who visited a psychosomatic clinic.

## 2. Methods

### 2.1. Setting

The psychosomatic outpatient clinic visited by patients in the present study was located in a tertiary-care hospital affiliated with a university in Tokyo [13,14]. The hospital includes 22 departments, and approximately 600,000 outpatients visit annually; the hospital has been described in detail previously [13,14]. The psychosomatic outpatient clinic, established in 2001, features two full-time staff members (a professor and an assistant professor) and one part-time member.

### 2.2. Subjects

We used data from a previous clinical trial, whose design was described in greater detail in a previous article [14]. Briefly, the subjects examined in the present study were outpatients visiting the psychosomatic clinic for the first time between April 2002 and March 2017. As described in our previous study [14,15], during their first visits, all patients underwent clinical interviews with physicians to obtain axis I diagnoses based on the Diagnostic and Statistical Manual of Mental Disorders, Fourth Edition, Text Revision (DSM-IV-TR) using a detailed diagnostic manual based on the Structured Clinical Interview for DSM-IV axis I disorders [16]. Each interview took approximately 30 min. To ensure diagnostic accuracy and improve reliability, all three physicians in the clinic met once weekly to discuss diagnoses [14]. The coded diagnoses were recorded in a database. The first and second diagnoses were identified when comorbid diseases were present [14]. However, only the first diagnosis was analyzed in the present study. Next, the patients were sorted into psychosomatic and non-psychosomatic (i.e., those with depression, anxiety, and other psychiatric disorders) groups [14], and only those in the psychosomatic group were analyzed in the present study. The psychosomatic group included those with medical conditions (code 316) and somatoform disorders (codes 300.11, 300.7, 300.81, 300.82, and 307.XX) affected by psychological factors.

With an approval of the Human Subjects Committee of the hospital, written informed consent was obtained from all participants.

### 2.3. Assessment of Psychosomatic Conditions

During the first visit, all patients completed the following four questionnaires after the clinical interview.

Medical Symptom Checklist (MSCL). Patients indicated the frequency, extent of discomfort, and degree of interference with daily activities of 23–25 medical symptoms listed in the MSCL. Based on previous studies on primary care patients [17,18] and our own work [19,20], the following 12 common major medical symptoms were selected for analysis: fatigue, headache, insomnia, back pain, abdominal pain, joint or limb pain, dizziness, chest pain, constipation, palpitation, nausea, and shortness of breath. Symptoms that occurred at least once per week were defined as “positive” symptoms, and the total number of somatic symptoms (0–12) was calculated by summing the number of such symptoms [19,20].

Twenty-item Toronto Alexithymia Scale. The TAS-20 is a self-report 20-item questionnaire assessing alexithymic characteristics using a five-point scale. The Cronbach’s alpha of the TAS-20 was 0.74 in a Japanese psychiatric outpatient sample [21]. The TAS-20 contains three subsets of questions (“factors”). The first factor, “difficulty identifying feelings,” includes seven items (e.g., “I am often confused about what emotion I am feeling”). The second factor, “difficulty describing feelings” includes five items (e.g., “It is difficult for me to find the right words for my feelings”). The third factor, “externally oriented thinking,” comprises the remaining eight items (e.g., “I prefer talking to people about their daily activities rather than their feelings”).

Somatosensory Amplification Scale (SSAS). The SSAS is a 10-item self-report questionnaire that assesses the tendency to amplify benign bodily sensations and experience them as noxious, unpleasant, and/or alarming; the SSAS employs an ordinal scale ranging from 1 to 5 [9]. A higher total score (range: 10–50) indicates greater symptom amplification. The Cronbach’s alpha of the SSAS was 0.79 in a Japanese outpatient sample [22].

Tokyo University Egogram (TEG). The reliability and validity of the questionnaire used to assess ego state, the TEG (60 items), has been tested in a Japanese population [23]. The TEG, which is based on transactional analysis theory [24], explores the following five ego-state scales: critical parent, nurturing parent, adult, free child, and adapted child. In the present study, the adapted child ego state was used to quantify the adaptive level; a person in the adapted child state might subordinate his or her own needs to those of others and behave so as to meet the expectations of others.

Profile of Mood States (POMS). The POMS is a 65-item questionnaire that assesses six mood states [25]; the reliability and validity of the Japanese version of the POMS have been established [26]. We analyzed the POMS scores for depression (range: 0–60) and tension–anxiety (range: 0–36). A higher score indicates greater depression or anxiety.

Stress Perception Scale. The degree to which patients reported stress in seven areas of life was evaluated using the self-report Stress Perception Scale [19,20]. The seven areas, which are evaluated using a 10-point scale (1 = no stress to 10 = worst stress possible), include work, family, and neighborhood relationships and living, social, financial, and health-related situations. Total scores are calculated by summing the scores of the seven areas. The Stress Perception Scale was used to assess the psychosocial stressors experienced by patients in the present study.

### 2.4. Data Analysis

Student’s *t*-test was used to compare continuous variables between females and males. The six variables selected for prediction of the total number of somatic symptoms were the scores on the TAS, SSAS, Stress Perception Scale, two POMS scales, and the TEG adapted child scale. After simple correlations between the total number of somatic symptoms and the selected variables were analyzed using Pearson correlational analyses, and multiple regression analysis was performed with the total number of somatic symptoms as the dependent variable and all six selected predictors, as well as age and sex, as independent variables. For reference, structural equation models were constructed to estimate the causal relationship between the total number of somatic symptoms and the six selected variables. Statistical significance was assessed using two-tailed *p* < 0.05. All statistical analyses were performed using the SAS ver. 9.4 statistical package (SAS Institute Inc., Cary, NC, USA) and SPSS Statistics Version 24 (IBM, Armonk, NY, USA).

## 3. Results

Patients’ basic characteristics are presented in Table 1. The average number of somatic symptoms was 4.8; fatigue (75.3%) was the most common symptom, followed by insomnia (56.1%), low-back pain (49.5%), headache (44.7%), and palpitations (43.1%). The average score on the Stress Perception Scale was 24.6; health concerns scored highest (5.9), followed by work (4.1), family (4.0), living (3.2), and financial situations (3.1). In terms of gender differences, the total number of somatic symptoms and the SSAS and POMS depression scale scores were significantly higher in females than in males.

**Figure 1:**
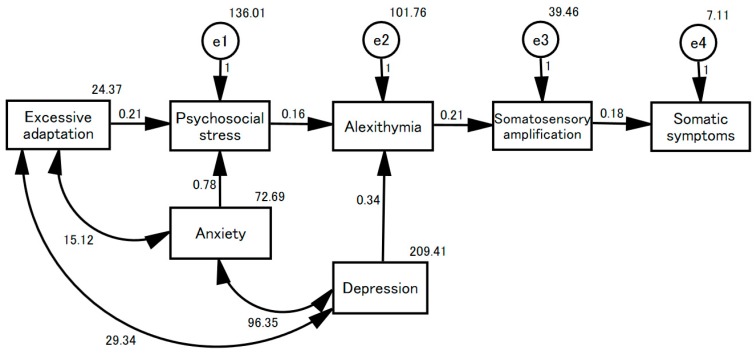
A model of somatic manifestation of psychosomatic illness. This structural equation model examines seven clinical variables in 604 psychosomatic outpatients. “Excessive adaptation” as reflected on the TEG Adapted Child scale; “anxiety” as scored on the POMS tension–anxiety scale; “depression” as revealed by the POMS depression scale; “psychosocial stress” as scored on the Stress Perception Scale; “alexithymia” as revealed by the TAS-20; and “somatosensory amplification” as shown by the SSAS. “Somatic symptoms”: the total number of somatic symptoms.

The associations among the seven clinical variables are presented in Table 2. All variables were significantly intercorrelated, even after controlling for the effects of age and sex. The results of multiple regression analysis are presented in Table 3. The total number of somatic symptoms was significantly associated with the TAS-20, SSAS, Stress Perception Scale, and POMS depression scale scores. A model linking psychosocial stress to somatic symptoms is proposed in Figure 1 based on the results of the structural equation model (goodness of fit (GFI): 0.882; adjusted GFI: 0.724; root mean square error of approximation (RMSEA): 0.206). The Stress Perception Scale score was explained by both the POMS Tension–Anxiety Scale and the TEG Adapted Child Scale. The TAS-20 score was explained by both the POMS depression scale and the Stress Perception Scale. The POMS Depression and Tension–Anxiety Scale scores and the TEG Adapted Child Scale score were correlated. The SSAS score was explained by the TAS score, and the total number of somatic symptoms by the SSAS score.

## 4. Discussion

We evaluated over 600 psychosomatic outpatients concerning physical, psychological, and stress-related conditions. The reported number of somatic symptoms was significantly related to somatosensory amplification, depression, and anxiety and to stress-related conditions, such as alexithymia, environmental adaptation, and perceived psychosocial stress. On multiple regression analyses, somatosensory amplification, depression, alexithymia, and perceived psychosocial stress were all significant and independent predictors of somatic symptoms.

As in our previous study [9], somatosensory amplification and alexithymia were closely correlated on both simple and partial correlation analyses adjusted by age and sex. When somatosensory amplification and alexithymia were compared in terms of their relationships to the reported number of somatic symptoms, the coefficients between somatosensory amplification and the reported number of somatic symptoms were relatively higher than those between alexithymia and symptom number on both simple and partial correlation analyses and multiple regression analysis. The coefficients between somatosensory amplification and perceived psychosocial stress were relatively lower than those between alexithymia and stress on both the simple and partial correlation analyses. These results support the idea that pathogenesis caused by stress perception, alexithymia, and somatosensory amplification explains somatic symptoms, as revealed by the structural equation model. Recently, alexithymia was reported to be positively associated with the number of somatic symptoms, as well as psychological factors, including somatosensory amplification and depression [27,28]. Future cohort studies will address the specific causal relationship between alexithymia and somatosensory amplification in somatizing and depressed patients.

In contrast, anxiety and adaptation were not significant contributors to somatic symptoms in the final multiple regression model, possibly because of collinearity problems [29]. On both simple and partial correlation analyses, anxiety was closely related to depression, and adaptation was related to both alexithymia and depression. However, it is interesting that both anxiety and adaptation explained stress perception in the structural equation model. Stress is regarded as the physiological and psychological reaction to circumstances that require behavioral adjustment, and anxiety as a biological warning system that prepares the body to either fight or flee in dangerous and stressful situations [19,30]. Thus, it may be that anxiety and adaptation, rather than being outcomes of the reported somatic symptoms, were attributable to stress perception when stress induced pathogenesis featuring somatic manifestations.

Several limitations existed in the present study. First, three different physicians performed the DSM-IV-TR diagnoses via clinical interviews to identify psychosomatic outpatients. Thus, inter-rater reliability may be a concern. To address the issue, as described in our previous study, we prepared a detailed diagnostic manual to minimize inconsistencies [12]. The second potential limitation was the generalizability of the results. It is important to consider the characteristics of our hospital, including its location. Additional studies in other Japanese institutions and non-Japanese hospitals are needed to improve the description of psychosomatic phenomena. The third limitation concerns inadequate model-fitting in the structural equation model [31]. In this model, neither the GFI nor AGFI attained >0.90, and the RMSEA was >0.10. Although the model should be carefully interpreted, the findings are clinically persuasive; residual factors affecting stress perception, alexithymia, somatosensory amplification, and reported somatic symptoms (i.e., e1–e4 in Figure 1) will be investigated in the next step of our work.

In spite of these limitations, the findings have several important practical implications. Psychosomatic medicine should focus on the bio-psycho-social aspects of health [32], as demonstrated in the present study. Not only statistically, but also clinically, it makes sense that alexithymia enhances somatosensory amplification making somatic symptoms more likely to develop. The roles of somatosensory amplification in the clinic should be further studied to clarify the pathogenesis of psychosomatic illness [33].

## Figures and Tables

**Table 1:** Clinical data from 604 outpatients defined as having “psychosomatic illness” in a Japanese psychosomatic clinic.

	Gender-Specific Scores			
	Total (*n* = 604)	Women (*n* = 401)	Men (*n* = 203)	*p* Values *
Total number of somatic symptoms, number	4.8 (2.9)	5.0 (2.9)	4.4 (2.9)	0.021
20-itemed Toronto alexithymia scale, scores	58.4 (11.9)	58.6 (12.2)	57.9 (11.3)	0.477
Somatosensory amplification scale, scores	30.9 (6.8)	31.5 (6.6)	29.7 (6.9)	0.002
Self-rating stress perception scale, scores	24.6 (13.6)	24.9 (13.3)	24.1 (14.0)	0.499
Depression scale on the profile of mood state, scores	25.2 (14.5)	26.0 (14.8)	23.4 (13.7)	0.038
Tension-anxiety scale on the profile of mood state, scores	19.2 (8.5)	19.5 (8.7)	18.6 (8.3)	0.234
Adapted child scale on the Tokyo University Egogram, scores	11.0 (4.9)	11.2 (5.1)	10.6 (4.6)	0.164

Data are shown as mean (standard deviation). * Each clinical variable was compared between females and males using Student’s *t*-test.

**Table 2:** Correlations of the total number of somatic symptoms (No. Symptoms) with scores on the 20-item Toronto Alexithymia Scale (TAS-20), Somatosensory Amplification Scale (SSAS), Stress Perception Scale (Stress Perception), the Profile of Mood States depression scale) (Depression), tension–anxiety scale (the Profile of Mood States anxiety scale) (Anxiety), and the Adapted Child scale of the Tokyo University Egogram (Adaptation).

	No. Symptoms	TAS-20	SSAS	Stress Perception	Depression	Anxiety	Adaptation
No. Symptoms	-	0.344	0.409	0.495	0.437	0.415	0.164
TAS-20	0.345	-	0.373	0.407	0.508	0.435	0.416
SSAS	0.403	0.350	-	0.378	0.393	0.464	0.291
Stress perception	0.492	0.386	0.365	-	0.546	0.508	0.235
Depression	0.434	0.483	0.372	0.545	-	0.781	0.411
Anxiety	0.411	0.409	0.499	0.503	0.770	-	0.359
Adaptation	0.152	0.383	0.268	0.220	0.386	0.388	-

Each value in the upper triangle is a Pearson correlation coefficient, and each in the lower triangle a partial correlational coefficient after controlling for the effects of age and sex. All associations featured *p*-values < 0.001.

**Table 3:** Prediction of the total number of somatic symptoms: results of univariate and multivariate analyses (*n* = 604).

	Multiple Regression Analysis	
	Standardized Regression Coefficients	*p* Values
Age, years	0.034	0.328
Sex (women = 1; men = 0)	0.048	0.159
Clinical variables, scores		
20-itemed Toronto alexithymia scale	0.090	0.031
Somatosensory amplification scale	0.205	<0.001
Self-rating stress perception scale	0.298	<0.001
Depression scale on the profile of mood state	0.144	0.014
Tension-anxiety scale on the profile of mood state	0.045	0.421
Adapted child scale on the Tokyo University Egogram	–0.074	0.055
